# In silico discovery of a FOXM1 driven embryonal signaling pathway in therapy resistant neuroblastoma tumors

**DOI:** 10.1038/s41598-018-35868-5

**Published:** 2018-11-30

**Authors:** Suzanne Vanhauwaert, Bieke Decaesteker, Sara De Brouwer, Carina Leonelli, Kaat Durinck, Pieter Mestdagh, Jo Vandesompele, Karen Sermon, Geertrui Denecker, Christophe Van Neste, Frank Speleman, Katleen De Preter

**Affiliations:** 1 Center for Medical Genetics (CMGG), Ghent University, Ghent, Belgium; 2 Cancer Research Institute Ghent (CRIG), Ghent University, Ghent, Belgium; 3 Research Group Reproduction and Genetics, Faculty of Medicine and Pharmacy, Vrije Universiteit Brussel, Laarbeeklaan 103, 1090 Brussels, Belgium

**Keywords:** Cancer genomics, Data mining, Prognostic markers, Paediatric cancer, Tumour biomarkers

## Abstract

Chemotherapy resistance is responsible for high mortality rates in neuroblastoma. *MYCN*, an oncogenic driver in neuroblastoma, controls pluripotency genes including LIN28B. We hypothesized that enhanced embryonic stem cell (ESC) gene regulatory programs could mark tumors with high pluripotency capacity and subsequently increased risk for therapy failure. An ESC miRNA signature was established based on publicly available data. In addition, an ESC mRNA signature was generated including the 500 protein coding genes with the highest positive expression correlation with the ESC miRNA signature score in 200 neuroblastomas. High ESC m(i)RNA expression signature scores were significantly correlated with poor neuroblastoma patient outcome specifically in the subgroup of *MYCN* amplified tumors and stage 4 nonamplified tumors. Further data-mining identified FOXM1, as the major predicted driver of this ESC signature, controlling a large set of genes implicated in cell cycle control and DNA damage response. Of further interest, re-analysis of published data showed that MYCN transcriptionally activates FOXM1 in neuroblastoma cells. In conclusion, a novel ESC m(i)RNA signature stratifies neuroblastomas with poor prognosis, enabling the identification of therapy-resistant tumors. The finding that this signature is strongly FOXM1 driven, warrants for drug design targeted at FOXM1 or key components controlling this pathway.

## Introduction

Childhood cancers have been considered as developmental disorders and differ in many aspects from adult cancers, including an undifferentiated cellular phenotype and a remarkable low mutational burden^1^. Embryonal tumors such as medulloblastoma, Wilms’ tumor, embryonal rhabdomyosarcoma and neuroblastoma arise due to disruption of normal early developmental pathways in immature progenitor cells causing differentiation arrest and formation of pre-malignant lesions that can subsequently develop to full blown tumors that retain distinct stemness characteristics^1,2^.

Neuroblastomas that arise mainly in very young children with a median age at diagnosis of 18 months, are believed to emerge from cells of the developing adreno-sympathetic nervous system^1,3^. In an earlier study, we provided support for this hypothesis through analysis of human fetal adrenal neuroblast transcriptomes which showed close similarity to malignant neuroblastoma gene expression profiles^4^. Also the few neuroblastoma driver genes that were identified so far, including *MYCN*, *ALK*, *PHOX2B* and *LIN28B*, are all involved in early stages of the sympathetic nervous system development^5–7^ with both LIN28B and MYCN implicated as major drivers of stemness^8^. Novel therapies targeting these neuroblastoma driver genes such as ALK inhibitors and inhibitors of the BRD4-MYCN-promotor interaction are already emerging^9^. While these developments are promising, outcome of high-risk neuroblastoma patients is still disappointingly low and insights into therapy resistance mechanisms and new venues for targeting these resistant cells are needed. High treatment failure rates could possibly be explained by the stemness features of cancer initiating cells, including enhanced DNA repair capacity^10^. Therefore, a more in-depth analysis of stemness features of pediatric cancers may provide new insights into therapy resistance and may also offer novel targets for therapeutic intervention.

In this study, we explored the stemness features of neuroblastoma tumor cells using an *in silico* analysis starting from a normal embryonal stem cell (ESC) driven miRNA signature. This approach was based on three important concepts. First, several hallmark characteristics of stem cells, including the capacity to self-renewal and differentiation are mimicked in the highly proliferative cancer cells, suggesting that similar regulatory networks are active in normal stem cells and cancer stem cells^11^. Secondly, miRNAs are key players in the tight control of (stem) cell fate^12^. These small non-coding RNA molecules play a crucial role in stem cell pluripotency, control of self-renewal, lineage-specific differentiation, and cell reprogramming^12^. The miRNA pathway has been shown to be crucial in embryonic development and in embryonic stem cells, as shown by Dicer knockout analysis^13^. Specific patterns of miRNAs have been reported to be expressed only in ESCs and in early phases of embryonic development. Hence, regulatory networks tightly controlled by miRNAs can be assumed to play important roles in normal and cancer stem cell biology. Third, miRNA expression patterns can distinguish tumor types and tissue types better than mRNA expression patterns^14^. From these observations we anticipated that miRNAs could act as a proxy to capture stem cell features in neuroblastoma cells. Indeed, it has already been shown that miRNAs play important roles in neuroblastoma tumor formation and progression^15,16^. The *let-7* cluster, previously described as crucial to be downregulated for maintaining pluripotency in stem cells^17^, is frequently lost in neuroblastoma, inversely associated with *MYCN* amplification status and independently correlated with poor prognosis^18^. Furthermore, several miRNAs have been shown to play a role in chemoresistance in neuroblastoma^19^.

To this end, we used a unique *in silico* approach to investigate the stemness characteristics in neuroblastoma tumor cells and obtained three important results: (1) a 60 miRNA ESC signature could discriminate patients with poor survival within a subset of the high-risk neuroblastoma patients; (2) biological function enrichment analysis on the coding genes correlated with the miRNA ESC signature score in neuroblastoma revealed increased DNA repair mechanisms in tumors with high stem cell capabilities driven by the FOXM1 transcription factor, which was further confirmed by FOXM1 knock down experiments; (3) analysis of transcriptional changes after FOXM1 knock-down in neuroblastoma cells and re-analysis of published datasets strongly supports the contribution of FOXM1 to the stemness and MYCN driven tumor phenotype of neuroblastoma cells. The results of these *in silico* analyses should fuel renewed efforts for identifying novel on target compounds to block FOXM1 activation in high-risk neuroblastomas.

## Results

### Embryonic stem cell miRNA signature score analysis identifies patients with poor survival within a subset of high-risk neuroblastoma patients

In order to evaluate the stem cell features of neuroblastoma cells, we first established a robust miRNA ESC signature based on literature data. To this end, gene lists were retrieved from 4 published studies reporting on differential miRNA expression analysis of ESCs versus more differentiated cells (Supplemental Table 1)^20–23^. miRNA genes that were listed in at least 2 of the publications were included in the ESC signature (Table 1). A gene cluster with a well-known role in ESCs is the miR-302/367 cluster, of which all components are present in the signature. Using signature score analysis (see material and methods), we could validate the signature in an independent dataset of ESCs with high miRNA signature scores for the ESC samples compared to differentiated somatic tissue (Supplemental Fig. 1)^24^.

**Table 1:** ESC miRNA signature.

Upregulated in ESC	Downregulated in ESC
hsa-miR-141	hsa-let-7a
hsa-miR-148a	hsa-let-7e
hsa-miR-187	hsa-let-7f
hsa-miR-18a	hsa-let-7g
hsa-miR-18b	hsa-miR-100
hsa-miR-20a	hsa-miR-125a
hsa-miR-20b	hsa-miR-125b
hsa-miR-200c	hsa-miR-132
hsa-miR-19a	hsa-miR-137
hsa-miR-19b	hsa-miR-143
hsa-miR-302a	hsa-miR-145
hsa-miR-302astar	hsa-miR-152
hsa-miR-302b	hsa-miR-181a
hsa-miR-302bstar	hsa-miR-181b
hsa-miR-302c	hsa-miR-21
hsa-miR-302d	hsa-miR-22
hsa-miR-367	hsa-miR-222
hsa-miR-363	hsa-miR-23a
hsa-miR-363star	hsa-miR-23b
hsa-miR-372	hsa-miR-24
hsa-miR-498	hsa-miR-27a
hsa-miR-512-3p	hsa-miR-27b
hsa-miR-515-5p	hsa-miR-28
hsa-miR-517a	hsa-miR-29a
hsa-miR-517b	hsa-miR-376a
hsa-miR-518b	hsa-miR-495
hsa-miR-518c	hsa-miR-99a
hsa-miR-520f	
hsa-miR-520g	
hsa-miR-520h	
hsa-miR-524star	
hsa-miR-92b	
hsa-miR-96	

**Figure 1:**
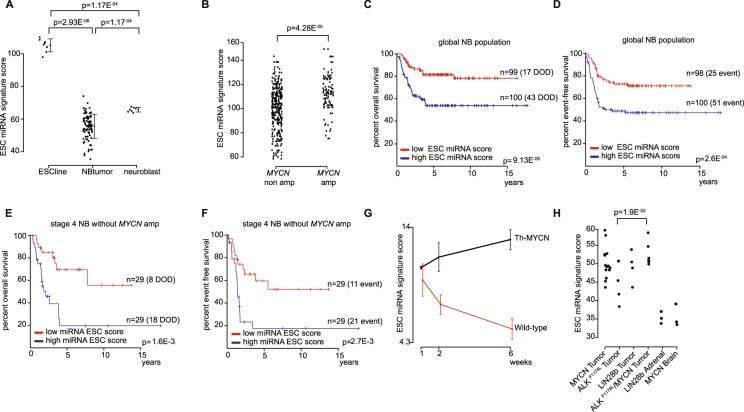
Identification of an ESC miRNA signature score that stratifies neuroblastoma patients within a subset of high-stage neuroblastoma. (**A**) ESC miRNA signature scores in embryonic stem cells (ESC line)^25^, neuroblastoma tumors (NB tumors)^26^ and normal neuroblasts^4^ (the error bars represent standard deviations, p-values from Mann-Whitney tests). (**B**) ESC miRNA signature scores in MYCN non amplified neuroblastoma tumors (MNA) compared to MYCN amplified neuroblastoma tumors (MA) (p-value from t-test). (**C,D**) Kaplan-Meier and log rank analysis of 200 neuroblastoma patients with a high or low ESC miRNA signature score (using median as cut-off). DOD, dead of disease. (**E,F**) Kaplan-Meier and log rank analysis within the subset of stage 4 MYCN non amplified patients. (**G**) ESC miRNA A signature score during tumor development in Th-MYCN transgenic mice. (**H**) ESC miRNA signature scores for MYCN, ALK^F1174L^ and Lin28b neuroblastoma mice tumors and their normal counterparts (adrenal gland and brain tissue) (p-value from Mann-Whitney test).

Next, we generated miRNA expression data of ESCs^25^, normal neuroblasts and neuroblastoma tumor samples^4,26^. We confirmed high scores for the ESCs compared to those of neuroblastoma tumor samples (Fig. 1A). The normal counterpart cells, i.e. the normal neuroblasts isolated from fetal adrenal glands, have ESC miRNA signature scores in the higher range of the scores identified in neuroblastoma tumors samples. The large dynamic range of scores in neuroblastoma tumors warranted us to evaluate whether this heterogeneous expression pattern could reflect tumor characteristics including *MYCN* status and patient survival. Indeed, tumors presenting with *MYCN* amplification have significantly higher signature scores than *MYCN* single copy tumors (t-test, p-value = 4.282e-05) (Fig. 1B). In addition, Kaplan-Meier and log-rank analysis pointed at a significant correlation with survival, i.e. patients with higher scores have lower survival chances (Fig. 1C,D). According to multivariate logistic regression analysis, this significant correlation is independent of the currently used risk markers, i.e. *MYCN* status, stage (stage 4 versus other stage) and age at diagnosis (below or above 1 year) (Odds’ ratio 3.08, p = 2.16E-2 for the signature score using median cut-off). More specifically, *MYCN* single copy stage 4 tumors with high ESC miRNA signature scores (above the median score) have extremely low survival probability, i.e. 19.6% overall survival at 5 years after diagnosis (95% confidence interval: 7.52–51.1%) versus 69.5% (52.6–91.9%) in tumors with low ESC miRNA signature scores (Fig. 1E,F).

Taken together, these data indicate that highly aggressive neuroblastoma cells in patients with ultra-high risk and very poor survival are enriched for a stemness ESC derived gene signature and that application of this signature could be helpful for early detection of patients for novel treatment strategies.

### The ESC miRNA signature score is dynamically upregulated during MYCN driven tumor formation and is highest in the ALK^F1174L^/MYCN double transgenic mice

In the tyrosine hydroxylase (Th)-MYCN neuroblastoma mouse model, a clear increase in the ESC miRNA signature score during tumor development from pre-neoplastic lesions in the ganglia at week 1 and 2 to full-blown tumors at week 6 after birth is observed (Fig. 1G)^27^. Furthermore, Th-*MYCN*- and also dopamine beta hydroxylase (dβh)-iCre; LSL- *ALK*^*F1174L*^-, and dβh-iCre; LSL-*LIN28B*-driven mice tumors are characterized by higher ESC miRNA signature scores compared to normal adrenal gland (Fig. 1H)^7,28^. Interestingly, the ESC miRNA signature score in double transgenic mice tumors (*MYCN/ALK*^*F1174L*^) is significantly higher than in *ALK*^*F1174L*^-driven tumors (significant difference with p = 1.905E-2), in concordance with shorter time to tumor appearance and thus more tumor aggressiveness in double transgenic mice compared to *ALK*^*F1174L*^-transgenic mice (Fig. 1H).

Overall, these results generated in human and mice tumors show that a high ESC signature score is linked to higher tumor aggressiveness, in keeping with the observed poor survival in patients with tumors with high miRNA ESC score.

### The ESC mRNA gene signature in neuroblastoma is highly enriched for FOXM1 driven cell cycle and DNA repair genes

**Figure 2:**
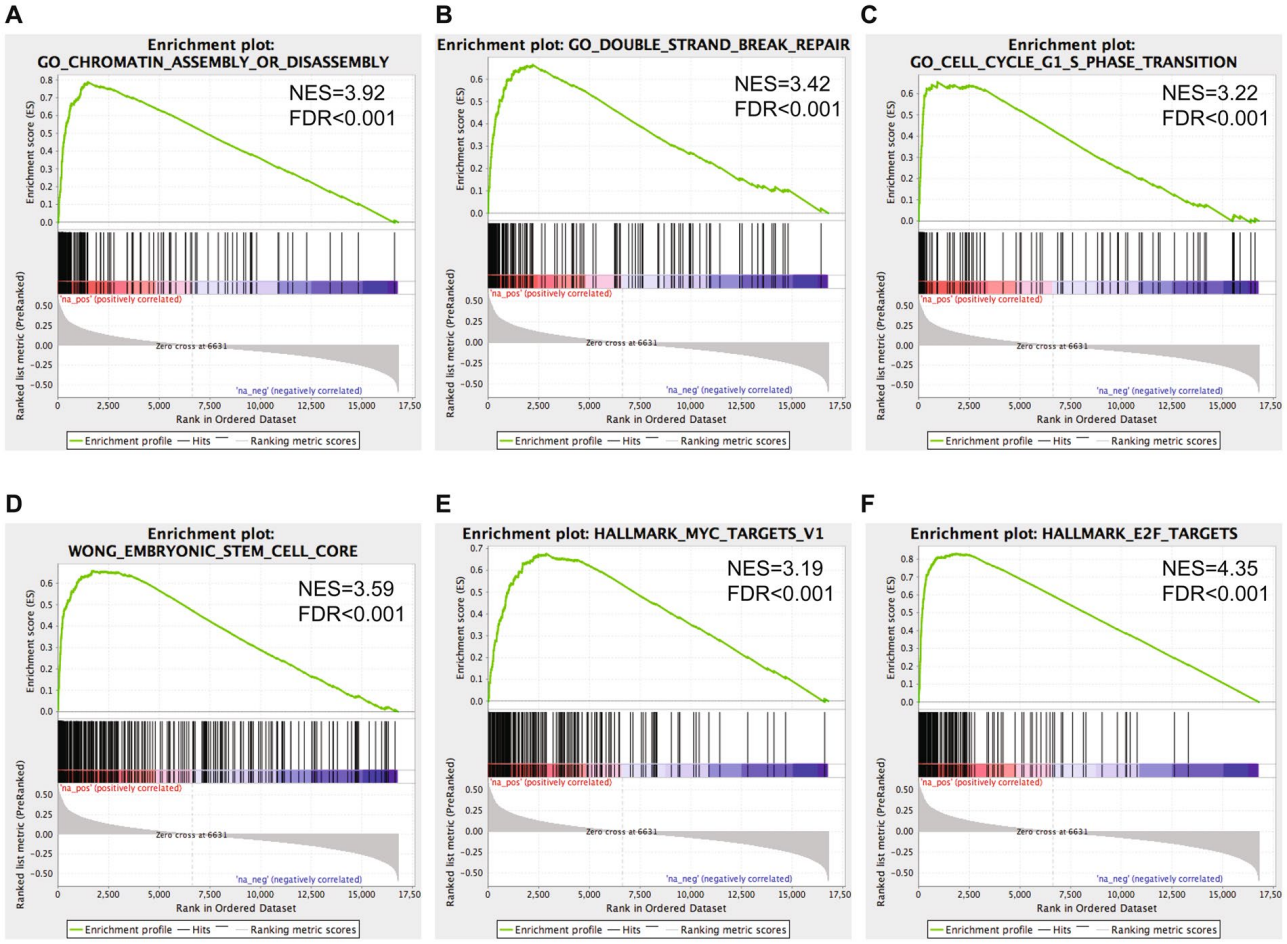
Important genesets found to be enriched using GSEA on the list of coding genes ranked according to the degree of correlation with the ESC miRNA signature score in 200 neuroblastoma patient samples. NES = nominal enrichment score; FDR = false discovery rate.

In order to understand the underlying biological functions of high miRNA ESC signature scores in aggressive neuroblastoma, Gene Set Enrichment Analysis (GSEA) analysis was performed on the list of coding genes ranked according to degree of expression correlation with the ESC miRNA signature score in 200 NB tumor samples (Supplemental Table 2). Gene sets enriched among the positively correlated genes are involved in chromatin remodeling, response to DNA damage (double strand DNA breaks) and cell cycle (MSigDB c5-gobp) as well as embryonic stem cells (MSigDB c2-cgp) and MYC and E2F targeting (MSigDB Hallmark genesets) (adjusted p-values < 0.001) (Fig. 2A–F). These findings were confirmed by DAVID-gene ontology analysis on the top 500 correlated genes showing enrichment for cell cycle and DNA damage/repair genes (functional annotation clustering: first cluster with cell cycle gene sets has enrichment score of 41.93, second cluster with DNA damage/repair functional classes has enrichment score of 29.52, cut-off for significance is 1.3).

We also performed motif enrichment analysis using iRegulon^29^ to identify transcription factors that drive the expression of the top 500 correlated genes, further referred to as the ESC mRNA signature (Supplemental Table 3)^29^. Within the top 10 most significantly enriched transcription factors, several members of the DREAM complex, which has an important role in transcriptional repression of cell cycle genes and maintaining quiescence, are present including E2F, MYBL2 and FOXM1^30^. Dream-complex members FOXM1, MYBL2, E2F1/2/3/7/8 and LIN9 are all part of the top 500 correlated gene list. Furthermore, FOXM1 is a transcription factor known to target several genes involved in the enriched DNA repair pathway, including *EXO1*, *BRIP1*, *BRCA1*, *BRCA2*, *CHEK1*, *BUB1B*, *XRCC2* and *RAD51AP1k*, all of which are in the top 50 of the ESC mRNA signature^31^. Also part of the gene list is *CENPF* which is a known FOXM1 target that cooperates with FOXM1 as recently shown for prostate cancer^32^, as well as *MELK*, *CDK6*, *PLK1* which are all described as regulators of FOXM1 phosphorylation^33^. Interestingly, the expression of 3 of these genes i.e. *MELK*, *MYBL2* and *PLK1* is remarkably highly correlated with *FOXM1* expression levels in 200 neuroblastoma tumors (correlation coefficients >0.9) suggesting a very tight co-regulated transcriptional control.

Furthermore, similar as to the ESC miRNA signature, ESC mRNA signature scores are related to survival in a global cohort of neuroblastoma tumors, but also in a subgroup of stage 4 tumors without *MYCN* amplification (Supplemental Fig. 2A–D). This observation is confirmed in an independent dataset of 498 neuroblastoma tumors (Supplemental Fig. 2E–H)^34^.

Collectively, our findings support that the ESC mRNA gene signature in neuroblastoma is also related to patient survival and most interestingly is highly enriched by components of a FOXM1 controlled regulatory network.

### Differentially expressed genes after FOXM1 knock-down in neuroblastoma cells are enriched for mRNA ESC signature genes

**Figure 3:**
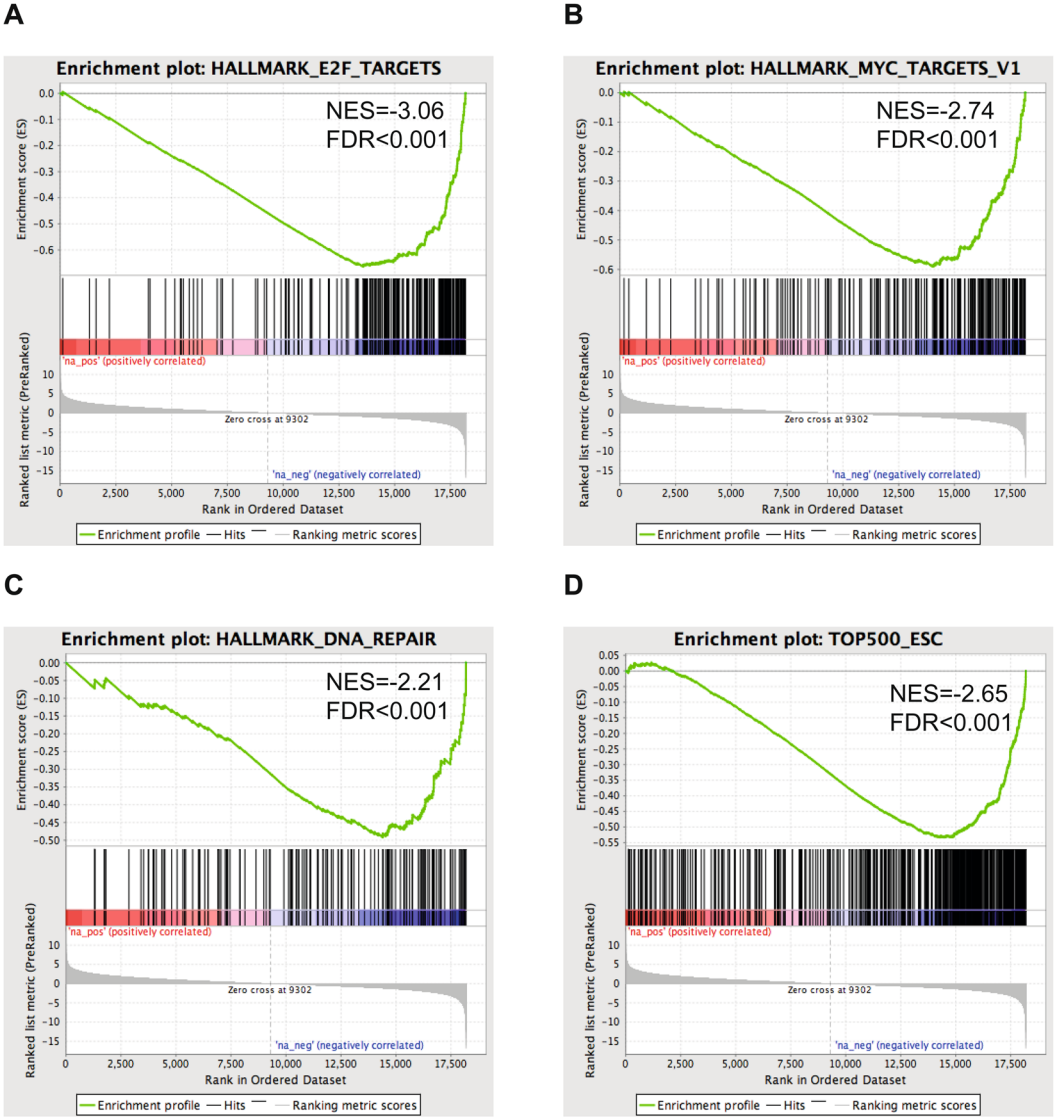
Important genesets found to be enriched using GSEA upon knock down of FOXM1 in IMR32. NES = nominal enrichment score; FDR = false discovery rate.

To investigate the effect of FOXM1 knock-down on the transcriptional profile and more particularly the ESC mRNA gene signature, we performed FOXM1 knock down and gene expression profiling in the neuroblastoma cell line IMR-32 with high ESC mRNA signature score and high *FOXM1* expression levels (4 biological replicates) (Supplemental Fig. 3A). Upon lentiviral transduction of shFOXM1 in IMR-32, we observed strong knockdown of FOXM1 expression levels (Supplemental Fig. 3B,C). In addition, genes downregulated upon shFOXM1 were significantly enriched for described FOXM1 direct target genes identified using ChIP-sequencing (Fisher’s Exact test p = 7.564e-15 and p = 2.758e-10)^35,36^. Subsequent GSEA of down regulated genes after FOXM1 knock-down revealed enrichment for gene sets related to DNA repair, E2F and MYC targeting (MSigDB Hallmark genesets) (Fig. 3A–C). Of further note, the 500 ESC gene set is also significantly enriched in the IMR-32 FOXM1 knock-down data (Fig. 3D) (GSEA-analysis, adjusted p-values < 0.001). This is also confirmed in other cancer types where the ESC mRNA signature goes down upon chemical and pharmacological FOXM1 knock-down (Supplemental Fig. 4)^37–39^.

Taken together, this experimental analysis further supports the data-mining driven observation that the ESC mRNA gene signature in neuroblastoma cells is enriched for genes that are transcriptionally regulated by FOXM1.

### In silico evidence for an activating role of MYCN on ESC genes in keeping with direct binding of MYCN to the FOXM1 promotor

**Figure 4:**
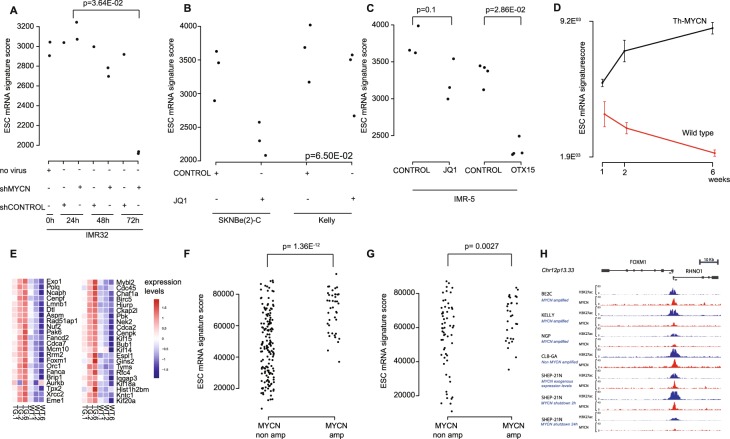
Association between MYCN and FOXM1 in the ESC mRNA signature score. (**A**) ESC mRNA signature score upon lentiviral knock-down of MYCN in IMR-32 neuroblastoma cells, (**B**) upon pharmacological inhibition of MYCN with JQ1 in SKNBE(2)-C and Kelly neuroblastoma cells, (**C**) after inhibition of MYCN with JQ1 and OTX015 in IMR-5 neuroblastoma cells (p-values from Mann-Whitney tests), and (**D**) during tumor development in Th-MYCN mouse model. (**E**) Heatmap of expression of the top 50 correlated genes of the ESC mRNA signature in the Th-MYCN mouse model. (**F,G**) ESC mRNA signature score in neuroblastoma patient samples with and without *MYCN* amplification in a global cohort (**F**) and in stage 4 neuroblastoma patient samples (**G**). (H) ChIP-seq profiles of H3K27 and MYCN transcription factor binding at the FOXM1 promotor in both MYCN amplified (NGP, BE2C, KELLY) and MYCN non amplified (CLB-GA, qval = 6.3 × 10^−5^) cell lines as well as the SHEP21N cell line with induced MYCN expression.

Previous work has provided evidence for a coordinated role of MYC and FOXM1 in controlling normal S-phase progression in mouse embryonic stem cells^40^. Furthermore, as indicated above, we observed MYC target gene enrichment in the genes downregulated upon FOXM1 knock-down. Therefore, we further investigated MYCN controlled regulation of the FOXM1 gene signature in neuroblastoma context. To this end, we reanalyzed data of several *in vitro* MYCN neuroblastoma model systems. In these datasets, we observed decreased ESC mRNA signature scores and *FOXM1* expression levels upon MYCN knock-down and pharmacological MYCN activity inhibition using JQ1 and OTX015 (Fig. 4A–C)^41–43^. Also in the Th-MYCN mouse model an increase in ESC mRNA signature score and *FOXM1* expression is observed upon *MYCN* driven development from hyperplastic ganglia towards full-blown neuroblastoma tumors (Fig. 4D)^27^. Remarkably, the expression of all top 50 correlated genes from the ESC mRNA signature, including *FOXM1*, increases upon Th-MYCN driven neuroblastoma development (Fig. 4E). In neuroblastoma tumors, the ESC mRNA signature score is significantly higher in tumors with versus without *MYCN* amplification (both in the global cohort and the subset of stage 4 tumors) (Fig. 4F,G Supplemental Fig. 2I,J). Interestingly, higher ESC mRNA signature scores were also observed in other tumor(cell line)s with *MYCN* amplification (Supplemental Fig. 5)^44,45^. Altogether, these data point to an important role of MYCN in the regulation of the ESC signature genes. Therefore, we further investigated the possible binding of MYCN to the FOXM1 promotor region using published ChIP sequencing data for MYCN^46^ and subsequent analysis of binding sites at or near the FOXM1 promotor. In these data, direct binding of MYCN on the FOXM1 promotor is clearly observed in both *MYCN* amplified and non-amplified high MYCN expressing cell lines (Fig. 4H). Strong MYCN binding promotors are highly enriched for the canonical E-box (CACGTG), and a canonical E-box motif is found at the transcription start site of FOXM1, indicative for a strong interaction between MYCN and the FOXM1 promotor. Also, MYCN binding to the strong promotor associated site of FOXM1 is only decreased 24 hours after MYCN shutdown while at other loci with weaker binding sites MYCN binding is lost already 2 hours after MYCN downregulation in SHEP-21N cells. This further shows that reduced binding of MYCN at the FOXM1 promotor only occurs when MYCN is already depleted from weaker MYCN binding sites. Altogether, these data suggest that MYCN acts a driver (or co-driver) of the FOXM1 controlled ESC signature.

## Discussion

Stem cell like (stemness) gene signatures have been shown to hold prognostic power in several cancer types^11^. Given that (1) neuroblastoma is an embryonal neural crest derived tumor, (2) MYCN is critical for the maintenance of embryonic stem cell-derived neural crest stem cells^8^ and (3) LIN28B, a known stem cell marker, acts as oncogene in neuroblastoma^7^, we decided to explore whether an ESC derived miRNA signature could capture a stemness phenotype in neuroblastoma cells and be applied as tool to select neuroblastoma patients with therapy resistant tumors. Having established an ESC derived miRNA prognostic classifier, we could indeed show that within the subgroup of high-risk patients we were able to predict with high confidence patients that would not benefit from current treatment regimes. Indeed, neuroblastoma patients with a high ESC miRNA signature score have more chance to die of disease or relapse as illustrated in Kaplan-Meier analyses. The survival difference for tumors with different scores is most striking for high-stage neuroblastoma tumors without *MYCN* amplification, while all tumors with *MYCN* amplification have higher ESC miRNA signature scores compared to non-amplified tumors. This prognostic classifier could thus be helpful to identify patients in a subset of high-stage cases that would be eligible for phase I trials for novel compounds in so-called basket trials^47^.

To understand the key drivers of the high ESC signature scores and possible cause of therapy resistance mechanisms in ultra-high risk neuroblastomas and as a prelude to drugging the stemness phenotype in neuroblastoma, we applied a unique data mining approach. We established mRNA signature derived from correlation analysis of the initial ESC miRNA signature score with gene expression profiling data in a large set of primary human neuroblastomas. Functional and motif enrichment analysis on the top 500 correlated genes, further referred to as the ESC mRNA signature, pointed at a central role of transcription factor FOXM1 and several other members of the DREAM complex, including MYBL2, E2F and LIN9 in controlling the stem cell characteristics of aggressive neuroblastoma cells, by keeping cell cycle and DNA repair mechanisms in check. The DREAM complex has an important role in cell cycle by coordinating the shift from quiescence to proliferation^30^. When cells exit the G0 phase, FOXM1 and MYBL2 are recruited to promote mitotic gene expression^30^ thereby controlling proper DNA replication and avoiding excessive genomic damage^30^. Binding of FOXM1 to G2/M promotors is dependent upon MYBL2, further revealing a tight interaction between both proteins^48^. MYBL2 has also been shown to be a downstream target of MYCN in neuroblastoma and the cells are addicted to MYBL2 in a MYCN dependent manner^49^. Both genes have been described to have a role in sustaining the self-renewal capacity of pluripotent stem cells^40^, and renewal of neural progenitor cells and survival of neuroblastoma cells^50^. In further support of a role for FOXM1 in neuroblastoma, Zha *et al*. reported high MEIS2 levels which is a transcription activator of the MuvB-BMYB-FOXM1 complex with FOXM1 being a direct target gene of MEIS2 and required for MEIS2 to upregulate mitotic genes^51^.

Given our findings and these data from the literature, we propose this regulatory axis as an important novel therapeutic vulnerability for neuroblastoma.

Next, we further explored the link between MYCN and stemness^8^ through further analysis of several MYCN model systems, i.e. the dynamic regulated transcriptome of Th-MYCN driven mouse neuroblastomas^27^, neuroblastoma cell lines upon knock-down of MYCN expression^41^ and upon pharmacological inhibition of MYCN activity^42,43^. Collectively, these data demonstrate correlation of high ESC mRNA signature scores with high MYCN activity, further confirmed by high ESC mRNA signature scores in MYCN amplified human tumors compared to non-MYCN amplified cases. Not unexpectedly, a subset of high-stage neuroblastoma tumors without *MYCN* amplification also have elevated ESC signature scores, in keeping with high MYC activity in these cells. Similar as with the miRNA ESC signature, this subgroup of neuroblastoma patients is marked by very poor survival outcome.

Interestingly, our ESC mRNA gene set shows a very strong overlap with a gene set recently derived by Olsen *et al*.^52^ in a novel mouse neural crest derived neuroblastoma model. In brief, this study describes how mouse neural crest cells are grown and differentiated *in vitro* to generate sympathetic progenitor cells. Subsequently, these cells were transduced with MYCN and injected into mice to generate neuroblastomas. Analysis of the MYCN upregulated genes in the resulting mouse tumors also showed a strong enrichment of FOXM1 controlled genes involved in cell cycle and DNA damage control corroborating our data in human primary tumors and the Th-MYCN mouse model.

In a huge pan-cancer dataset including ~18.000 tumors, a *FOXM1* regulatory network was identified as a major predictor of adverse outcome across different tumor entities^53^, matching with the described link of FOXM1 with cancer therapy resistance^54^ and its role in DNA damage control^31^ as observed in stem cells and tumors like neuroblastoma. In the light of the studies that pinpoint FOXM1 as an important pan-cancer gene, several labs are undertaken efforts to identify drugs that target FOXM1. The past years, several drugs presumed to target FOXM1 activity have been reported but so far none of these drugs has been successfully applied in the clinic, possibly due to off target and or insufficient on target effects of these drugs. While our study warrants further drug screening for more potent and specific FOXM1 inhibitors^39^, it is also of great interest that the FOXM1 upstream regulatory kinase MELK is also highly expressed, suggesting that this could act as a good candidate drug target for ultra-high risk neuroblastoma patients. More recently, a specific MELK inhibitor, MELK-T1 was tested, and could represent a useful novel drug for improving survival rates for children with neuroblastoma^55^.

Similar as our ESC miRNA signature, Ittai Ben-Porath *et al*.^11^ has built an ESC mRNA signature consisting of 380 genes overexpressed in ESC according to 5 or more out of 20 profiling studies and could show an association to prognosis in breast cancer. We tested this mRNA signature in expression data of 200 neuroblastoma tumors and could show correlation of high signature score with worse outcome in the global patient cohort, but not in the subset of high-stage tumors without *MYCN* amplification (data not shown). Moreover, the signature did not contain the DREAM complex genes, FOXM1, MYBL2, LIN9 and E2F. Thus, our unique approach did not only lead to more strong and robust prognostic signatures, but also revealed some putative mechanism of therapy resistance in neuroblastoma.

In conclusion, by (re)analysis of published and unpublished expression profiling data, we could unravel a MYC(N)-FOXM1-ESC signaling axis that is active in therapy resistant neuroblastoma tumors and that unveils new vulnerable nodes for targeted therapy of tumors where current treatment regimens fail.

## Materials and Methods

### Re-analysis of publicly available datasets

In this study, we reanalyzed several published expression datasets: miRNA expression data of different embryonal stem cell lines and differentiated tissue (GSE34199)^20–24^, 2) miRNA expression data of 200 neuroblastoma tumor samples^26^, 3) matching mRNA expression profiling data of the same 200 samples (GEO85047), 4) RNA sequencing data of 498 neuroblastoma tumors (GSE62564, GSE49711)^34^, 5) miRNA and mRNA expression data of the Th-MYCN neuroblastoma progression model (E-MTAB-2618)^27^, 6) miRNA expression data of the different neuroblastoma mouse models^7,27^, 7) mRNA expression data of the shMYCN knock-down system (GSE39218)^41^, 8) mRNA expression data of JQ1 and OTX015 treated neuroblastoma cell lines (GSE43392, E-MTAB-3672)^42,43^, 9) mRNA expression profiling data of siFOXM1 treated breast cancer cells (GSE55204, GSE25741)^56,57^ and shFOXM1 treated glioma cells (GSE63963)^58^, 10) mRNA expression profiling data of glioma, prostate, and breast cancer cell lines after pharmacological inhibition of FOXM1 with siomycinA and FDI-6 (GSE50227, GSE36531, GSE58626)^37–39^, 11) data of the cancer cell line encyclopedia^59^ and 12) medulloblastoma mRNA expression data (GSE30530)^45^. The R-package GEOquery and ArrayExpress were used to download the publicly available (normalized) data directly into the R-environment. ChIP-seq data of MYCN and H3K27ac in BE2C, Kelly, NGP and SHEP21N were downloaded from ArrayExpress (E-GEOD-80154) and converted to bigwig files to visualize the ChIP tracks in IGV.

### miRNA profiling of normal neuroblasts and human embryonic stem cell lines

MiRNA expression of 8 embryonal stem cell lines^25^ and 7 normal neuroblast samples^4^ was profiled and normalized as previously described^60^ (data submitted to a data repository (accession number GSE121513).

### Data-mining and statistical analysis

We performed signature score analysis on both mRNA and miRNA expression data using a rank-scoring algorithm as described in^61^. In brief, for each tumor sample (mRNA or miRNA) expression values were transformed to ranks (a rank of 1 matching with the lowest expressing gene). Next, rank scores for the signature genes were summed for each sample generating a signature score.

Correlation of the score with survival was tested using Kaplan-Meier plots and log-rank analysis by grouping the samples in 2 equal groups (score above or below the median value) (R-survival package). Comparison of signature scores or expression between groups of samples was done using the parametric t-test or non-parametric Mann-Whitney test (R-base package). Signature score and gene expression correlation analysis was performed using Pearson correlation analysis (R-base package). GSEA^62^ was performed using the version 5.2 geneset catalogue. Multivariate logistic regression analysis was performed on a subset of samples including only the patients that died of disease or survived for at least 3 years after diagnosis.

### FOXM1 gene silencing through shRNA knock-down and verification of knock down by RT-qPCR and Western Blot analysis

ShRNA knock down for FOXM1 was achieved using MISSION shRNA (Sigma) TRCN0000015544. Viral production was performed as instructed by the manufacturer (Lifetechnologies) and the obtained virus was afterwards transduced in the neuroblastoma cell line IMR-32.

24 h after transduction the medium was refreshed and cells were selected with puromycin (0.5 µg/ml). Cells were harvested for RNA 96 h after transduction and RNA isolation was performed using the miRNeasy micro kit (Qiagen, catalogue number 217084), including DNAse treatment on column (RNAse-free DNAse set, Qiagen, catalogue number 79254). cDNA synthesis was carried out using 500 ng of RNA with the iScript cDNA synthesis kit (Bio-Rad, catalogue number 170–8891). RT-qPCR primers for FOXM1 (AGACACCCATTAAGGAAACG,TTTGTACTGGGCTGAAATCC) and reference genes HPRT1(TGACACTGGCAAAACAATGCA,GGTCCTTTTCACCAGCAAGCT), YWHAZ(ACTTTTGGTACATTGTGGCTTCAA, CCGCCAGGACAAACCAGTAT), SDHA (TGGGAACAAGAGGGCATCTG, CCACCACTGCATCAAATTCATG) were designed using primerXL (www.primerXL.org). RT-qPCR reactions were performed in duplicate in a total volume of 5 µl, including 2 µl of cDNA and 3 µl of ssAdvanced SYBR Green qPCR mastermix (Bio-Rad). Cycling conditions were 95 °C (15 s) – 60 °C (15 s) – 72 °C (60 s) and 44 cycles. Data analysis was performed using the qBasePlus software (Biogazelle). Protein extraction was done via RIPA buffer and protein concentration was measured using the Lowry protein assay. Protein extracts were separated with SDS-PAGE, blotted on a nitrocellulose membrane and probed with antibodies against FOXM1 (1/1000; 5436 S cell signaling), and β-actin (1/10000, A2228, Sigma-Aldrich). Proteins were detected with HRP-conjugated goat anti rabbit IgG antibody (1/15000, A27036, thermos fisher scientific) and developed with ChemiDoc-it imaging system (UVP).

### Differential gene expression analysis by RNA sequencing of FOXM1 knock-down in neuroblastoma cells

Poly-A captured RNA library preparation was done on biological quadruplicates of FOXM1 knock-down and control samples, using the TruSeq stranded mRNA kit LT. Concentration was measured via qPCR using the Kapa Library Quantification Kit (Illumina) and 1.4pM was loaded on a NextSeq. 500. NextSeq. 500 High Output V2 75 cycles kit was used for single end sequencing to obtain approximately 20 million reads for every sample.

Sample and read quality was checked with FastQC (v0.11.3). Reads were subsequently aligned to the human genome GRCh38 with STAR aligner (v2.5.2b). Final gene count values were obtained with RSEM (v1.2.31), which takes read mapping uncertainty into account.

Counts were normalized with the TMM method (R-edgeR package), followed by voom transformation and differential expression analysis with limma (R-limma package). GSEA^62^ was performed on the list ordered according to differential expression statistic value (t).

### ChIP-sequencing of MYCN and H3K27ac in neuroblastoma cell line CLB-GA

In addition to published ChIP-seq data, we also performed ChIP-seq in the non-MYCN amplified CLB-GA cell line. Chromatin immunoprecipitation for MYCN and H3K27ac was done in fifty million CLB-GA cells using 12.5 µg of MYCN-specific (Santa-Cruz, B8.4.B, sc-53993) and H3K27ac-specific (Abcam, ab4729) antibody according the ChIP-protocol described in^63^. DNA was subsequently adaptor ligated and amplified using the NebNext Ultra DNA Library Prep Kit (E7370S) and sequenced on the NextSeq. 500 using the NextSeq. 500 High Output Kit V2, 75 cycles kit (Illumina). Raw reads were mapped to hg19 reference genome using Bowtie2 and peakcalling was performed using MACS2. Bigwig files were generated to visualize the ChIP-seq tracks in IGV.

### Neuroblastoma cell lines

The CLB-GA cell line was obtained from the lab of Valerie Combaret (Lyon, France).

The IMR-32 cell line was obtained from the lab of Rogier Versteeg (Amsterdam, The Netherlands).

All cell lines were screened upon initiation in the lab with the MycoAlert Detection Assay (cat nr. LT07–318, Lonza) and immediately expanded for freezing in order to assure Mycoplasma free cells as a stock. Routinely monthly random screenings are done, which are each time completely negative. CLB-GA and IMR32 were passaged two times prior to the experiments. All cell lines were routinely STR genotyped, to ensure the authenticity of the cell lines.

## Electronic supplementary material

Supplementary Dataset 3

Supplementary Dataset 1

Supplementary Dataset 2

Supplementary information
