# Publisher Correction: In silico discovery of a FOXM1 driven embryonal signaling pathway in therapy resistant neuroblastoma tumors

**DOI:** 10.1038/s41598-019-44435-5

**Published:** 2019-06-04

**Authors:** Suzanne Vanhauwaert, Bieke Decaesteker, Sara De Brouwer, Carina Leonelli, Kaat Durinck, Pieter Mestdagh, Jo Vandesompele, Karen Sermon, Geertrui Denecker, Christophe Van Neste, Frank Speleman, Katleen De Preter

**Affiliations:** 1 Center for Medical Genetics (CMGG), Ghent University, Ghent, Belgium; 2 Cancer Research Institute Ghent (CRIG), Ghent University, Ghent, Belgium; 3 Research Group Reproduction and Genetics, Faculty of Medicine and Pharmacy, Vrije Universiteit Brussel, Laarbeeklaan 103, 1090 Brussels, Belgium

Correction to: *Scientific Reports* 10.1038/s41598-018-35868-5, published online 30 November 2018

In the original version of this Article, the author Katleen De Preter was incorrectly indexed. This error has now been corrected.

