# Supplementary material for: In silico discovery of a FOXM1 driven embryonal signaling pathway in therapy resistant neuroblastoma tumors
Source: Sci Rep. 2018 Nov 30;8:17468. doi: 10.1038/s41598-018-35868-5 (PMC6269481; doi:10.1038/s41598-018-35868-5)

SUPPLEMENTAL FIGURES

In silico discovery of a FOXM1 driven embryonal signaling pathway in therapy resistant neuroblastoma tumors

Authors: Suzanne Vanhauwaert, Bieke Decaesteker, Sara De Brouwer, Carina Leonelli, Kaat Durinck, Pieter Mestdagh, Jo Vandesompele, Karen Sermon, Geertrui Denecker, Christophe Vanneste, Frank Speleman, Katleen De Preter

---

Supplemental figure 1: Validation of the miRNA ESC signature in an independent dataset

(p-value from Mann-Whitney test)

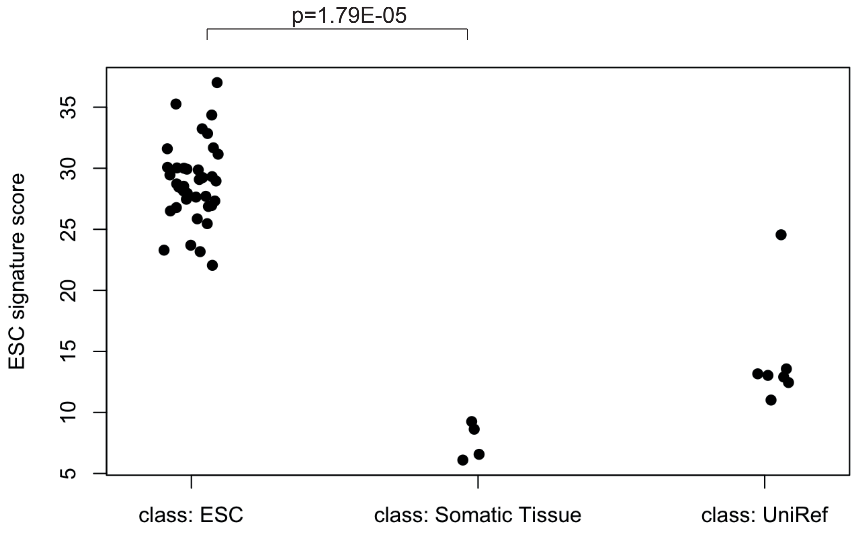

Supplemental figure 2: ESC mRNA signature score is related to survival

(A-B) Kaplan-Meier and log rank analysis on 200 neuroblastoma patients with a high or low ESC mRNA signature score. (C-D) Kaplan-Meier and log rank analysis within the subset of stage 4 neuroblastoma without MYCN amplification. Kaplan-Meier and log rank analysis of 498 neuroblastoma patients with high or low ESC mRNA signature score (independent validation), in the global cohort (E-F) and with the subset of stage 4 neuroblastoma without MYCN amplification (G-H). (I-J) ESC mRNA signature score in neuroblastoma patients with or without MYCN amplification in the global cohort and stage 4 neuroblastomas only.

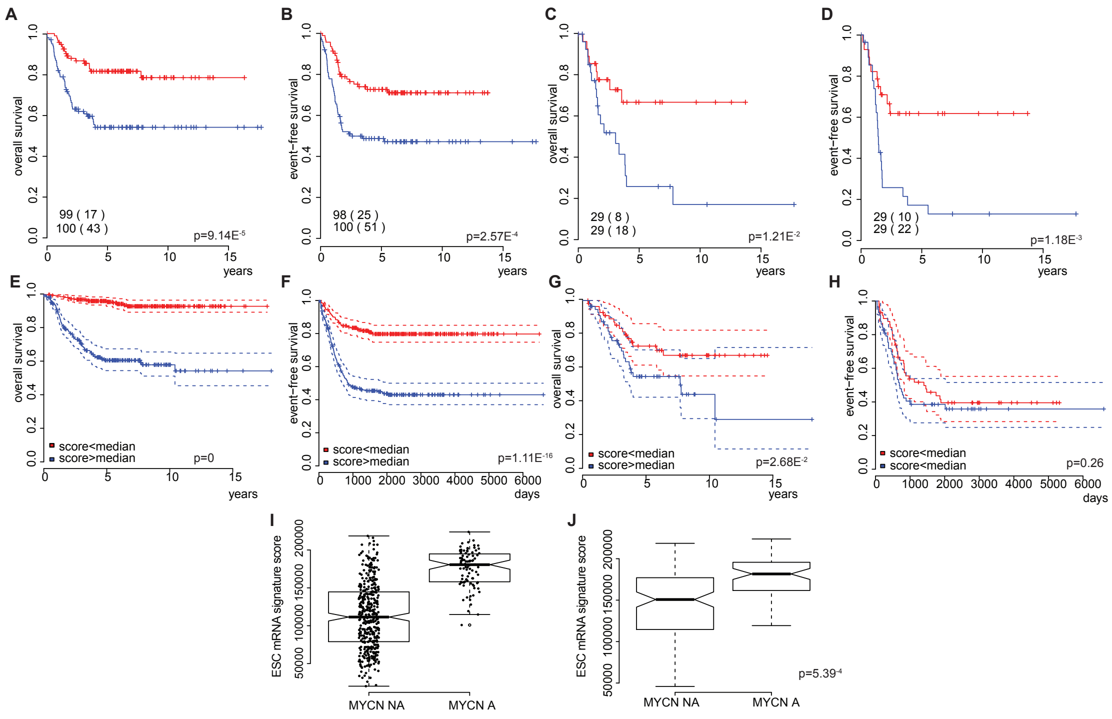

Supplemental figure 3: Lentiviral transductions of FOXM1

(A) ESC mRNA signature score and FOXM1 expression levels in 29 neuroblastoma cell lines. (B) Western Blot results for FOXM1 knock down with shRNA (loading control tubulin), (C) relative FOXM1 mRNA expression levels for FOXM1 knockdown with shRNA and control.

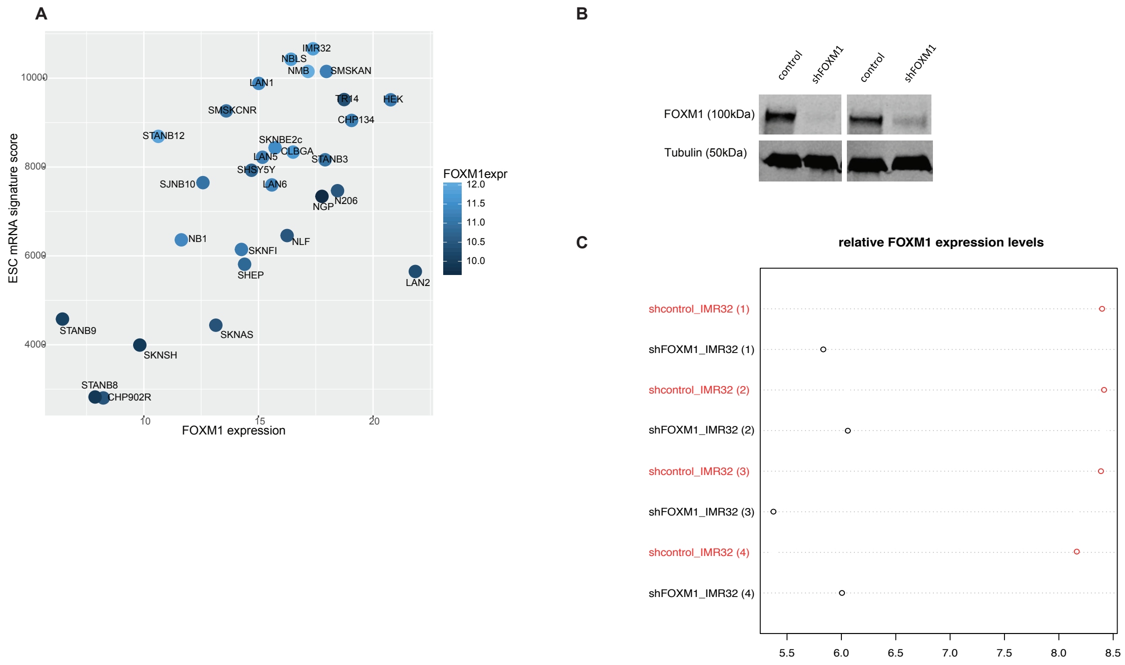

Supplemental figure 4: FOXM1 also drives the ESC mRNA signature score in other cancer entities

A) ESC mRNA signature score upon lentiviral inhibition of FOXM1 in glioma cells, (B-C) upon siRNA FOXM1 inhibition in breast cancer cells, (D) upon inhibition of FOXM1 with siomycin A in glioma stem cells, (E) upon inhibition of FOXM1 with siomycin A in prostate cancer cells and (F) in breast cancer cells treated with FDI-6 to inhibit FOXM1.

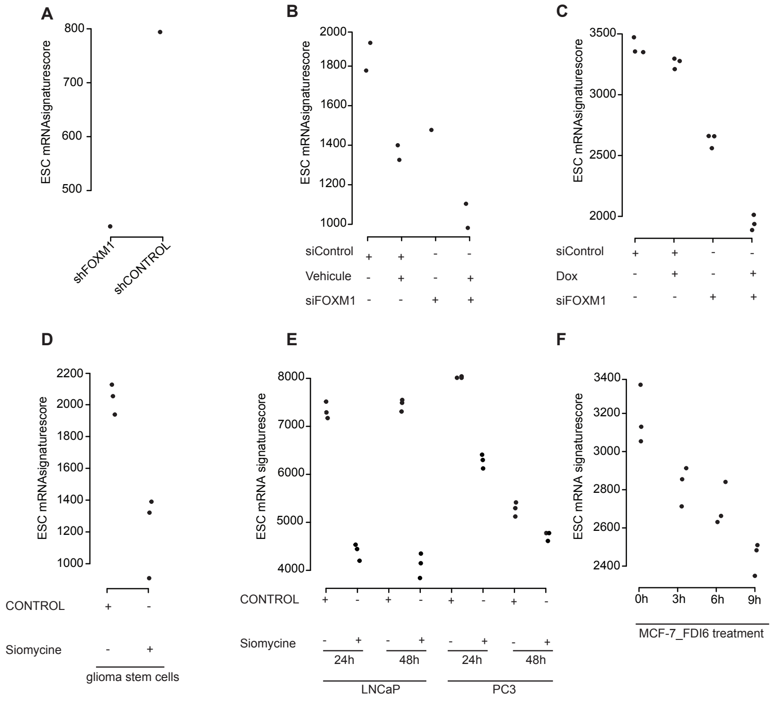

Supplemental figure 5: ESC mRNA signature scores in other cancer entities with or without *MYCN* amplification

A) CCLE database analysis of the ESC mRNA signatures in *MYCN* non-amplified and *MYCN* amplified cancer cell lines. (B) ESC mRNA signature scores in medulloblastoma samples with and without *MYCN* amplification (p-values from t-tests).

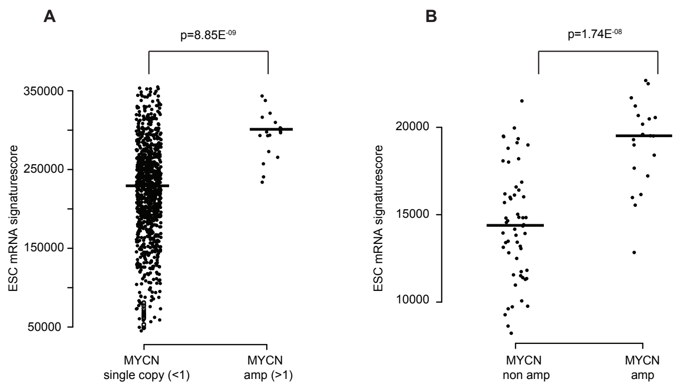

Supplement: Supplementary file 4 — Supplementary information [file 41598_2018_35868_MOESM4_ESM.pdf]
